# Gait adaptations and spinal curvature correlations following lumbar arthrodesis: an analytic cross-sectional study

**DOI:** 10.1186/s12891-026-10264-7

**Published:** 2026-08-06

**Authors:** Alessia Putzolu, Guillaume Claus, Arnaud Delafontaine, Clara Leyh, Laurent Fabeck, Véronique Feipel

**Affiliations:** 1 Laboratoire d’Anatomie, de Biomécanique et d’Organogenèse, Faculté de Médecine, Université Libre de Bruxelles, Route de Lennik 808, Bruxelles, CP 619 - 1070 Belgique; 2 Laboratoire d’Anatomie Fonctionnelle, Faculté des Sciences de la Motricité Humaine, Université Libre de Bruxelles, Route de Lennik 808, Bruxelles, CP 619 – 1070 Belgique; 3 CIAMS, Université Paris-Saclay, Orsay, 91404 France

**Keywords:** Chronic low back pain, Spinal curvature, Spine surgery, Lumbar arthrodesis, Gait analysis

## Abstract

**Background:**

Chronic low back pain is a major and growing public health concern worldwide. When conservative management fails, lumbar spinal fusion (arthrodesis) is a common surgical option. While postoperative pain outcomes have been extensively studied, the functional impact of lumbar arthrodesis on posture and gait remains poorly understood.

This study aimed to analyze gait characteristics in patients following lumbar arthrodesis and to examine correlations between gait parameters and spinal curvature changes.

**Methods:**

We conducted an analytic cross-sectional study including two groups: healthy participants and individuals who had undergone lumbar arthrodesis. Spinal curvatures were assessed using an optoelectronic stereophotogrammetric system, which recorded marker positions placed on a brace worn by the examiner during spinal palpation (A-palp method). Gait parameters were measured as participants walked at slow, fast, and self-selected speeds on an instrumented walkway equipped with pressure sensors.

**Results:**

Thirteen participants with lumbar arthrodesis were compared with fifteen healthy controls. Significant differences were observed between groups in gait parameters, particularly stride length, stride width and foot progression angle. In healthy participants, lumbar lordosis showed correlations with several gait parameters, whereas in the arthrodesis group correlations were mainly observed with foot progression angle.

**Conclusions:**

Individuals with lumbar arthrodesis demonstrated differences in selected gait parameters compared with healthy controls whereas no significant differences were observed in lumbar lordosis or thoracic kyphosis. Correlations between spinal curvature and gait parameters were mostly observed in healthy subjects, with weaker associations also observed in the arthrodesis group. Further longitudinal studies are needed to characterize gait patterns before and after lumbar arthrodesis and to determine whether these differences are related to surgery, chronic low back pain, or other patient-related factors.

## Introduction

Chronic low back pain (CLBP) is a major public health concern worldwide, leading to substantial personal, social, and economic burdens. Expenditures on analgesic treatments, including opioids and epidural corticosteroid injections, as well as costs associated with diagnostic imaging, have markedly increased over recent decades [1]. The chronic nature of pain adversely affects patients’ functional capacities and psychological well-being, making daily activities challenging and contributing to workplace absenteeism [2, 3].

CLBP, defined as pain persisting for more than three months [2, 3], can be classified as degenerative, non-degenerative, or idiopathic in origin [1, 3]. Degenerative causes include disc disorders, facet joint osteoarthritis, spondylolysis, and spondylolisthesis, often leading to radiculopathies and spinal stenosis with radiating symptoms, paresthesia, or functional impairment [3]. Non-degenerative etiologies encompass inflammatory, infectious, neoplastic, and traumatic pathologies [1, 3].

First-line management typically involves analgesic medications, particularly nonsteroidal anti-inflammatory drugs [3–5], while second-line pharmacological options may include opioids, tricyclic antidepressants, muscle relaxants, and gabapentin [4–6]. Non-pharmacological treatments such as physiotherapy, psychotherapy, acupuncture, ultrasound therapy, lumbar braces and orthoses, transcutaneous electrical nerve stimulation, and yoga can serve as adjuncts or alternatives [6, 7]. Among invasive non-surgical interventions, epidural or intra-facet corticosteroid injections and radiofrequency nerve ablation are commonly employed [4, 8].

Orthopedic insoles have demonstrated improvements in low back pain for patients with pronated feet, which can induce structural and biomechanical deficits during gait affecting the lumbar region [9, 10]. However, their efficacy across all etiologies of low back pain remains uncertain [9–12].

When conservative approaches fail, surgical intervention may be considered. According to the American Pain Society, surgical management may be proposed, outside of emergency cases such as cauda equina syndrome or significant motor deficits, when degenerative pathology persists despite at least one year of appropriate conservative care [1, 3]. Lumbar spinal fusion (i.e. posterior arthrodesis) and disc replacement are the most commonly performed procedures.

Lumbar arthrodesis immobilizes one or more intervertebral joints to reduce pain, instability, and deformity [13, 14]. While numerous studies report improvements in pain, disability perception, and overall quality of life following fusion [14–17], evidence regarding functional outcomes, particularly gait characteristics, remains limited. Postoperative improvements in pain-free walking distance, walking speed, and step length have been observed [18], yet comparisons with conservative management have not consistently demonstrated significant differences [14]. Postoperative disadvantages, including persistent pain, dissatisfaction, spinal stiffness, limited trunk flexion, and paraspinal muscle atrophy, have also been reported, especially after multi-level fusions [13, 18, 19].

Despite the clinical importance of functional outcomes, the impact of lumbar arthrodesis on gait and posture remains insufficiently characterized, with most studies limited to assessments of spinal immobilization [13, 20]. The present study aims to analyse gait patterns in individuals who have undergone lumbar arthrodesis, evaluate their correlations with spinal curvature changes, and improve the understanding of functional adaptations following lumbar arthrodesis. To our knowledge, no previous study has comprehensively addressed this topic.

## Methods

### Study design

This cross-sectional study included participants, divided into two groups: a patient group (5 females, 8 males; mean age: 50.7 ± 8.7 years; body mass index (BMI) 28.5 ± 3.0 kg/m^2^), comprising thirteen individuals who had undergone lumbar arthrodesis, and a control group of fifteen non-fused healthy subjects (6 females, 9 males; mean age: 52.5 ± 10.0 years; BMI 27,6 ± 3.0 kg/m^2^). Among the thirteen participants in the arthrodesis group, all had undergone instrumented lumbar arthrodesis. Seven patients had undergone single-level arthrodesis and six had undergone two-level arthrodesis. Clinical information regarding postoperative pain intensity, disability scores, medication use, and rehabilitation protocols was not consistently available and could therefore not be included in the analyses. Patient group participants were recruited on a voluntary basis from the Spine Clinic of the University Hospital Center Saint-Pierre, in Brussels, Belgium. Control participants were recruited among personal acquaintances. All participants were recruited between May 2021 and March 2022.

Given the lack of published studies investigating both spinal curvature and gait parameters in patients following lumbar arthrodesis using a comparable methodology, reliable effect size estimates were not available to perform an informative a priori power analysis. In line with recommendations, the study was considered exploratory, and sample size was determined based on feasibility constraints, with a convenience sampling approach [21, 22].

### Exclusion criteria

Participants in both groups were excluded if they had a history of vestibular or orthostatic disorders, uncorrected visual impairments, postural disorders, or motor disabilities. Control participants with any prior spinal surgery or spinal pathology were also excluded. Participants reporting acute low back pain episodes in the three months preceding testing were additionally excluded.

### Ethics considerations

The study protocol was approved by the Ethics Committee of the University Hospital Center Saint-Pierre in Brussels, Belgium (reference number: CE/21-05-04). All participants received detailed information about the study and were free to decline participation or withdraw at any time. Written informed consent was obtained from all participants in line with the Declaration of Helsinki, following a clear explanation of the study’s objectives and procedures. Participant safety was monitored continuously during data collection and evaluations were stopped immediately in case of pain, discomfort, fatigue or instability.

### Experimental procedures

All assessments were conducted at the Laboratory of Functional Anatomy in Free University of Brussels. Following a brief anamnesis documenting surgical history for the patient group, a physical examination was performed for all participants, including recording age, sex, height, weight and body mass index.

These variables were reported descriptively when available but were not used for stratified subgroups analyses to avoid loss of statistical power.

Two experiments were then conducted to evaluate spinal curvatures and gait parameters.

#### Analysis of spinal curvatures

Spinal curvatures were measured using a repeatable and reproducible standardized protocol [23–25].

A VICON T40s motion capture system (14 cameras, 100 Hz) was used to acquire all three-dimensional coordinates. Data collection was carried out by two trained operators. According to established guidelines [26], reflective markers were placed on standardized anatomical landmarks of the lower limbs, pelvis, acromial region, selected spinal points and head, enabling the calculation of static alignment. During acquisition, participants stood in the center of the capture volume, arms hanging alongside the body, to improve between-subject consistency.

- A-palp device

For spinal digitization, the A-Palp method was used [23–25], which employs a fingertip-supported splint equipped with a cluster of four reflective markers. Prior to data collection, the examiner calibrated the fingertip on a reference plate [23–25] to standardize digitizing accuracy. Then, the examiner digitized six scapular landmarks: the inferior angle, trigonum spinae, anterior and posterior acromial angles, the acromioclavicular joint and the coracoid process, to establish a reliable shoulder-girdle reference frame. Spinal palpation was subsequently performed three times, tracing the spinous processes continuously from the external occipital protuberance to S2 vertebra, while the VICON system captured the three-dimensional trajectory of the fingertip device. Only sagittal-plane curvature data were retained for analysis.

b. Labelling and data processing

**Fig. 1:**
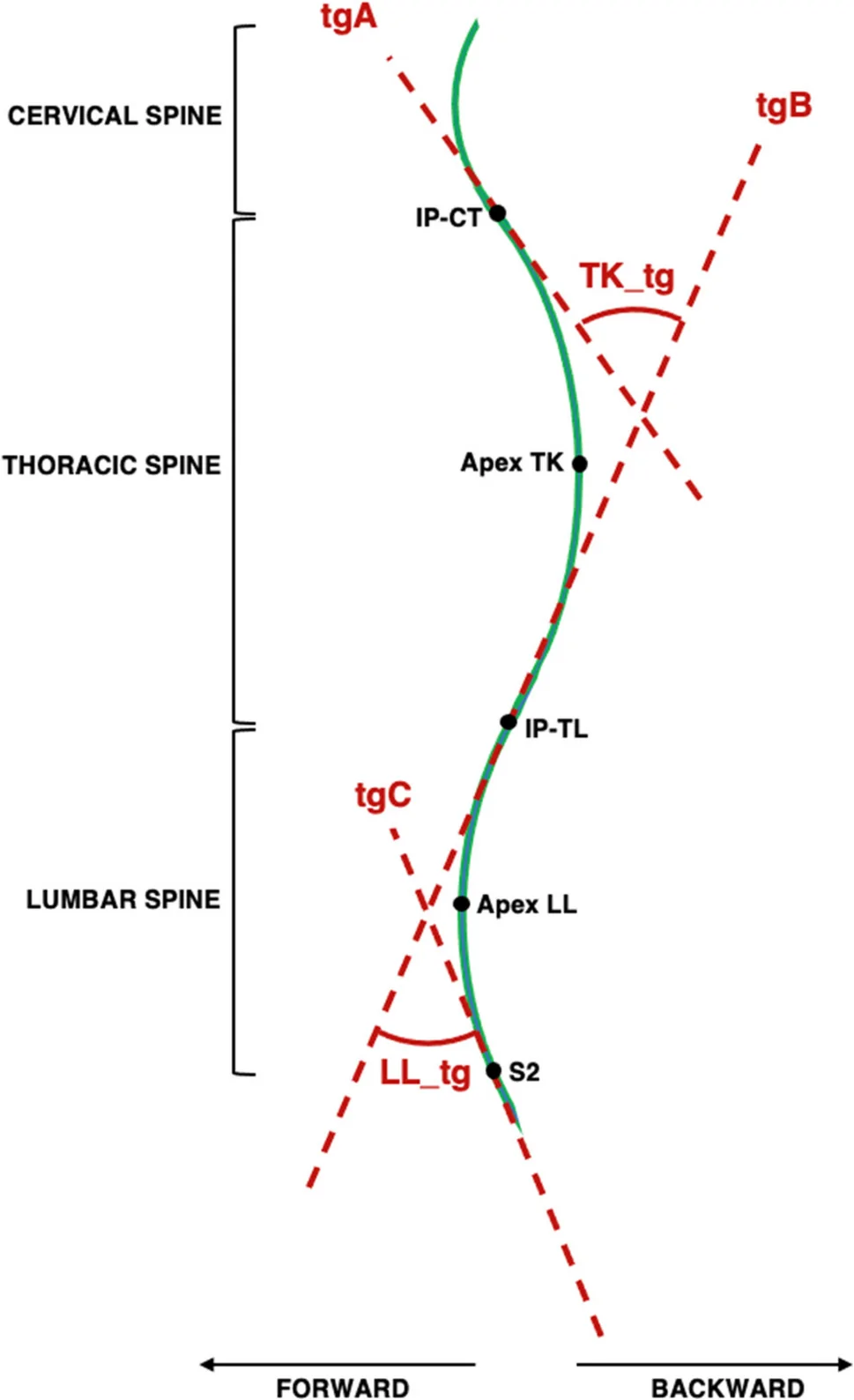
Illustration of sagittal spinal angle measured by the tangent method. Tangent method (red): In the thoracic region, the thoracic kyphosis angle TK_tg is computed between tangents at the cervicothoracic inflection point IP-CT (tgA) and the thoracolumbar inflection points IP-TL (tgB). Landmark: Apex TK = apex of thoracic kyphosis. In the lumbar region, lumbar lordosis angle LL_tg is computed between tangents at the thoracolumbar inflection points IP-TL (tgB) and S2 vertebra (tgC). Landmark: Apex LL = apex of lumbar lordosis

Reflective markers trajectories were manually labelled and gap-filled in Nexus 2.11 (VICON, Oxford, UK) using standard reconstruction procedures, then exported in C3D format for offline processing. The datasets were subsequently analysed with Biomechanical Toolkit (BTK) and custom MATLAB routines (R2019b) to isolate the calibrated fingertip trajectory obtained during palpation [26]. The three-dimensional path of the digitized spinous process was then resampled to 101 equally spaced points and smoothed by a procedure based on free-knot B-splines [27]. Two inflection points on the spinous process trajectory were manually selected. Using these boundaries, a local geometric model of the curve was generated by fitting on osculating circle according to Taubin’s method [28]. The resulting arc was then smoothly reintegrated into the full spinal trajectory through a modified AKIMA interpolation procedure. Based on the reconstructed three-dimensional coordinates, a dedicated MATLAB algorithm computed thoracic kyphosis (TK) and lumbar lordosis (LL) angles [29, 30] within the sagittal plane (Fig. 1) using the tangent method determined by the orientation of the tangent vectors evaluated at the inflection points, as defined by the Frenet frame at these locations.

#### Gait analysis protocol

Spatiotemporal gait parameters were measured using a validated protocol [26, 27, 31] with a Zeno walkway (6.10 m × 0.61 m, ProtoKinetics LLC, USA) integrating pressure sensors. To increase the likelihood of recording steady-state gait, participants were instructed to start walking approximately 2 m before the beginning of the instrumented walkway and to continue walking approximately 2 m beyond its end, thereby limiting the influence of acceleration and deceleration phases. Participants completed two round trips along the walkway at slow, fast, and self-selected speeds, totalling 12 passes. Data were processed using PKMAS software (5.07C8, ProtoKinetics Movement Analysis Software, ProtoKinetics LLC, USA) to identify left and right steps and remove artifacts. Extracted parameters included foot progression angle (°), step length (cm), stride length (cm), stride width (cm), stride velocity (cm/s), stance and swing phases (%), single and double support (%), walking speed (cm/s), and cadence (steps/min). Incomplete footfalls, steps partially outside the active recording surface, turning steps, and visually irregular footfalls related to acceleration, deceleration, or loss of rhythm were excluded before final parameter extraction.

### Statistical analysis

All data were exported in Microsoft Excel. Normality was assessed using the Shapiro-Wilk. Depending on distribution, two-tailed Student’s t-tests or Mann–Whitney U tests were performed to compare groups. Statistical significance was set at *p* < 0.05. Pearson’s correlation coefficient (r) was calculated to assess relationships between spinal curvature and gait parameters. Correlation strength was classified as strong (|r| > 0.75), moderate (0.75 > |r| > 0.50), weak (0.50 > |r| > 0.25), or absent (|r| < 0.25) [21]. Analyses were conducted using jamovi software version 2.7.30, released on 19 May 2026 (The jamovi project, Sydney, Australia).

## Results

### Spinal curvatures

Mean values of lumbar lordosis (LL) and thoracic kyphosis (TK) were compared between the control and patient groups. No statistically significant differences were observed. All results are summarized in Table 1.

**Table 1:** Comparison of spinal curvature values between groups

Variable (°)	Groups*		*p*-Value
	Control (*n* = 12)	Patient (*n* = 13)	
Lumbar lordosis	45 ± 12	41 ± 14	0.512
Thoracic Kyphosis	53 ± 8	46 ± 10	0.092

* : Data are expressed as mean ± standard deviation

### Gait analysis

Mean gait parameters of the *patient group* were compared to those of the *control group* for each walking speed (Table 2).

**Table 2:** Comparison of mean gait variables between groups

Variable	Speed	Groups*		*p*-value
		Control (*n* = 15)	Patients (*n* = 13)	
Foot progression angle (°)	Slow	5 ± 5	9 ± 5	0.060
	Preferred	5 ± 7	9 ± 5	0.109
	Fast	5 ± 4	9 ± 7	**0.046**
Stride length (cm)	Slow	113 ± 11	94 ± 17	**0.002**
	Preferred	128 ± 13	118 ± 14	**0.049**
	Fast	147 ± 10	129 ± 19	**0.006**
Stride width (cm)	Slow	12 ± 4	9 ± 4	0.071
	Preferred	12 ± 3	9 ± 3	**0.015**
	Fast	11 ± 3	9 ± 3	**0.021**
Stride velocity (cm/s)	Slow	80 ± 16	73 ± 27	0.372
	Preferred	109 ± 16	110 ± 22	0.783
	Fast	162 ± 17	140 ± 35	0.134
Stance phase (%)	Slow	65 ± 2	67 ± 5	0.333
	Preferred	63 ± 2	63 ± 2	0.496
	Fast	61 ± 2	62 ± 2	0.496
Single support (%)	Slow	35 ± 2	33 ± 5	0.185
	Preferred	37 ± 2	37 ± 2	0.730
	Fast	39 ± 1	38 ± 2	0.420
Double support (%)	Slow	30± 7	34 ± 10	0.316
	Preferred	26 ± 3	26 ± 4	0.821
	Fast	22 ± 2	24 ± 5	0.413
Walking speed (cm/s)	Slow	80 ± 16	73 ± 27	0.384
	Preferred	113 ± 16	110 ± 22	0.759
	Fast	155 ± 17	139 ± 35	0.135
Cadence (steps/min)	Slow	84 ± 13	90 ± 22	0.386
	Preferred	105 ± 7	111 ± 14	0.132
	Fast	126 ± 10	128 ± 17	0.805

*Data are expressed as mean ± standard deviation

*P *<0.05: Bold values indicate statistically significant results P

Significant differences were observed for foot progression angle at fast speed. Stride length differed significantly at all walking speeds. Stride width differed significantly at preferred and fast speeds. No other significant differences were observed

### Correlation between spinal curvature angles and gait parameters

Pearson’s correlation coefficients (r) were calculated between spinal curvature angles and gait parameters in both groups (Table 3).

**Table 3:** Pearson’s r correlation coefficients between lumbar lordosis, thoracic kyphosis and gait variables

Variable		Speed	Lumbar lordosis		Thoracic Kyphosis	
			Control(*n* = 15)	Patients(*n* = 13)	Control(*n* = 15)	Patients(*n* = 13)
Foot progression angle (°)		Slow	-0.37	-0.41	-0.62	-0.39
		Preferred	-0.09	-0.32	-0.02	-0.53
		Fast	-0.29	-0.25	-0.68	-0.34
Stride length (cm)		Slow	0.15	0.25	0.22	-0.10
		Preferred	-0.38	0.11	-0.07	0.03
		Fast	-0.36	-0.08	-0.04	0.03
Stride width (cm)		Slow	-0.11	-0.13	-0.58	0.32
		Preferred	0.02	-0.11	-0.66	0.32
		Fast	0.13	-0.23	-0.55	0.17
Stride velocity (cm/s)		Slow	-0,05	0.17	0.14	-0.17
		Preferred	-0,29	0.18	0.10	0.10
		Fast	-0.16	-0.07	0.19	-0.01
Stance phase (%)		Slow	0.26	-0.10	0.15	0.16
		Preferred	0.55	-0.18	0.38	-0.09
		Fast	0.12	-0.05	0.20	-0.06
Single support (%)		Slow	-0.13	0.11	-0.17	-0.14
		Preferred	-0.36	0.21	-0.27	0.13
		Fast	-0.19	0.06	-0.08	0.09
Double support (%)		Slow	0.20	-0.11	0.18	0.15
		Preferred	0.48	-0.19	0.34	-0.11
		Fast	0.15	-0.05	0.14	-0.07
Walking speed (cm/s)		Slow	-0.06	0.18	0.13	-0.17
		Preferred	-0.29	0.19	0.10	0.10
		Fast	0.16	-0.07	0.18	-0.01
Cadence (steps/min)		Slow	-0.13	0.05	0.06	-0.25
		Preferred	-0.04	0.22	0.26	0.18
		Fast	0.38	0.02	0.21	0.03

### Correlation between lumbar lordosis and gait parameters

**Fig. 2:**
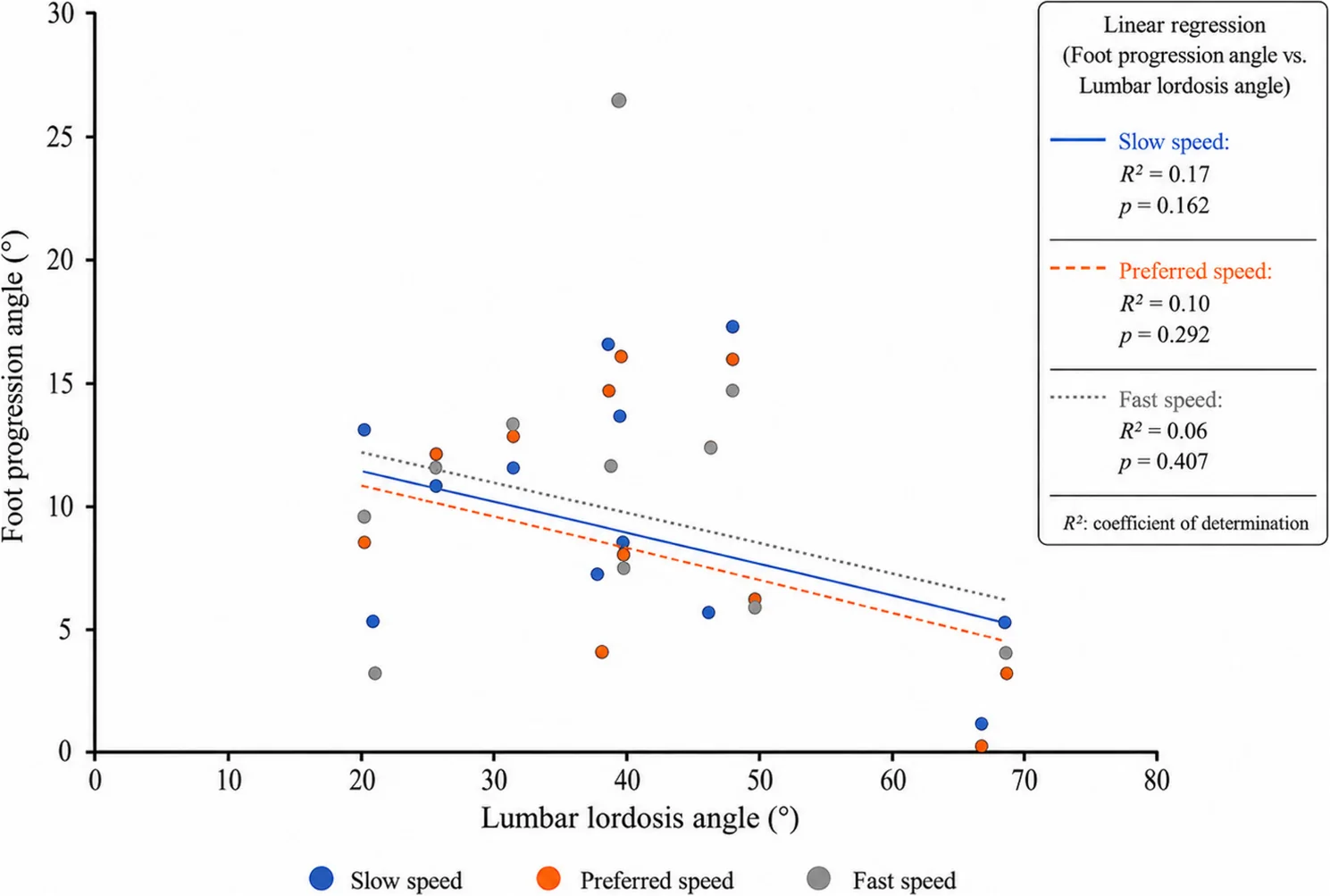
Correlation between Lumbar lordosis and foot progression angle in patient group

In the patient group, LL was weakly negatively correlated with foot progression angle at most walking speeds and with stride length at slow speed. In the control group, LL was moderately positively correlated with stance phase at preferred speed. LL was weakly positively correlated with double support at preferred speed and cadence at fast speed. It was weakly negatively correlated with foot progression angle at slow and fast speeds (Fig. 2), stride length at preferred and fast speeds; stride velocity, single support and walking speed at preferred speed.

### Correlation between thoracic kyphosis and gait parameters

**Fig. 3:**
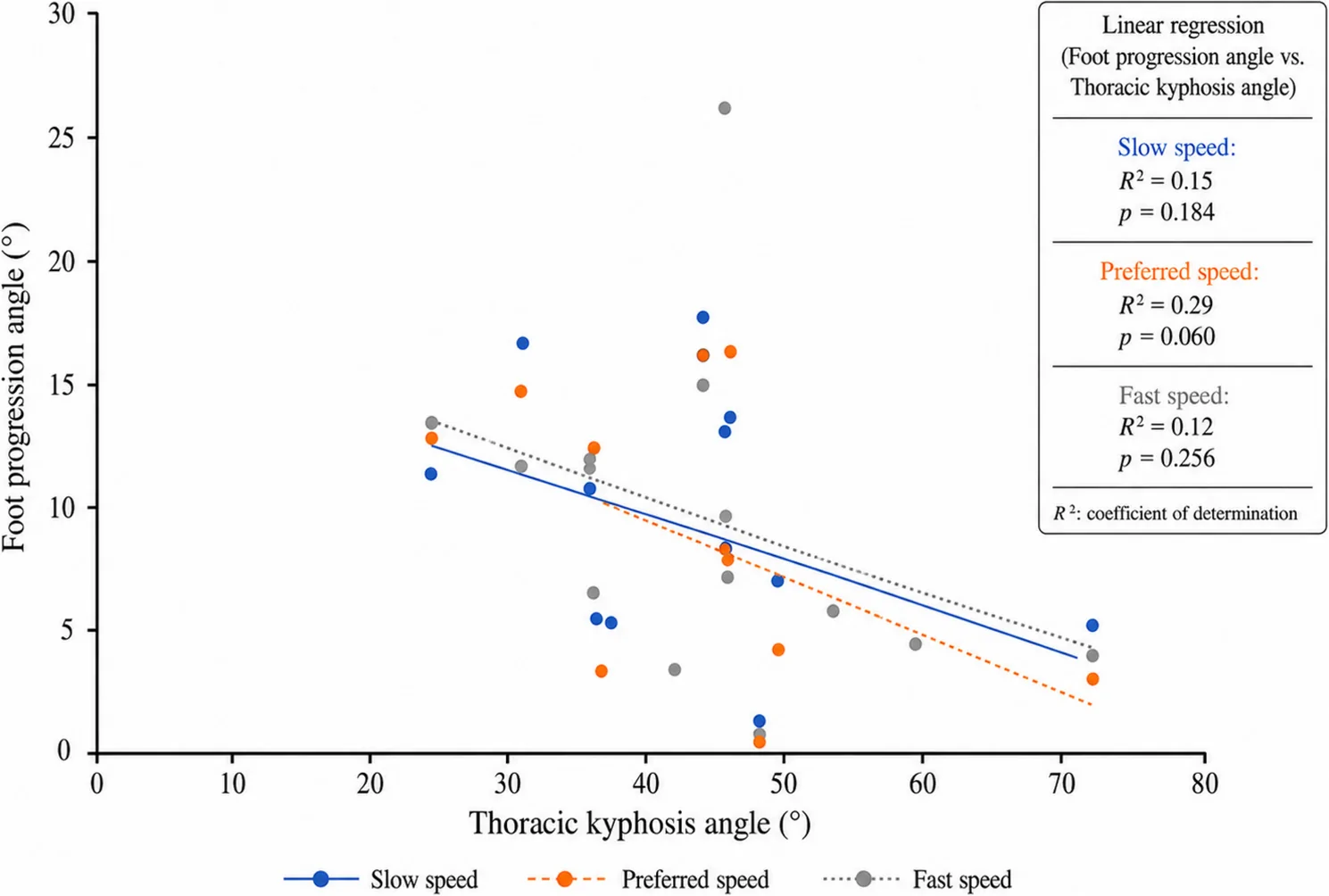
Correlation between thoracic kyphosis and foot progression angle in patient group

In the patient group, TK was moderately negatively correlated with foot progression angle at preferred speed (Fig. 3). It was weakly positively correlated with stride width at slow and preferred speeds; and negatively correlated with foot progression angle at slow and fast speeds.

In the control group, TK was moderately negatively correlated with stride width at all speeds and foot progression angle at slow and fast speeds. It was weakly positively correlated with cadence at preferred speed. It was weakly negatively correlated with single support at preferred speed.

## Discussion

Lumbar spinal arthrodesis is considered a treatment of last resort for chronic, degenerative, and disabling low back pain. While its effects on pain and disability are well documented, its functional impact, particularly on gait and posture, remains insufficiently explored. The present study aimed to analyze gait characteristics in patients following lumbar arthrodesis and to examine potential correlations with spinal curvatures.

In terms of spinal curvature, the arthrodesis group showed lower mean lumbar lordosis and lower mean thoracic kyphosis values than the control group. However, these differences did not reach statistical significance. Therefore, no firm conclusion can be drawn regarding between-group differences in sagittal spinal curvature in the present sample.

Interestingly, these findings are consistent with those reported by Tchachoua Jiembou et al. [32], who observed no significant restoration of lumbar lordosis following posterior lumbar arthrodesis despite marked improvements in pain, disability, and quality of life. Although they identified associations between lordosis restoration and clinical improvement in specific subgroups, their overall findings suggest that satisfactory postoperative outcomes may occur even in the absence of significant changes in lumbar lordosis [32]. In the present sample, our findings similarly indicate that postoperative lumbar lordosis should not necessarily be considered a surrogate marker of functional recovery.

Previous studies have emphasized the potential influence of lumbar fusion on spinopelvic sagittal alignment [33] and the interrelationship between thoracic kyphosis and lumbar lordosis within sagittal balance [34]. Nevertheless, considering the limited sample size and cross-sectional design of the present study, the observed numerical differences should be interpreted as descriptive findings only and should not be considered evidence of postoperative loss of spinal curvature.

Regarding gait parameters, patients with lumbar arthrodesis exhibited greater foot progression angle compared with controls. Stride length and stride width were reduced. These findings are partially consistent with gait patterns commonly reported in individuals with chronic low back pain, including shorter steps or strides and altered gait strategies [35–38]. These gait characteristics may reflect protective behavior aimed at minimizing lumbar loading and perceived instability. As shown in previous studies on chronic low back pain, individuals often exhibit a slower walking speed, shorter step (or stride) length, increased stance or support phases, and altered trunk-pelvis coordination, changes commonly interpreted as protective or compensatory strategies to reduce lumbar stress and perceived instability [39–42]. The reduced stride width observed in the arthrodesis group is more difficult to interpret. Although an increased step width is often considered a strategy to enhance mediolateral stability, a narrower base of support may instead reflect reduced pelvic excursion, limited trunk motion, or a more rigid gait pattern. Because mediolateral trunk and pelvic kinematics were not assessed, these interpretations remain speculative.

The increased foot progression angle observed in the arthrodesis group may reflect the contribution of multiple components of the locomotor system, including hip rotation, pelvic mechanics, lower-limb alignment and trunk control [43, 44]. Therefore, the mechanisms underlying this adaptation remain unclear and should not be interpreted as a direct marker of lumbar compensation alone.

Notably, differences between groups were most pronounced at preferred and fast walking speeds. At slow speeds, participants were generally able to compensate for postural instability [45], whereas higher speeds require greater adaptive strategies. Pain-related kinesiophobia may also contribute, prompting patients to reduce movement amplitude to maintain perceived safety [46–48]. Because psychological variables such as kinesiophobia or pain catastrophizing were not assessed in the present study, their contribution remains speculative. These findings suggest that gait characteristics observed in patients with a history of lumbar arthrodesis share similarities with those previously reported in chronic low back pain populations. However, it remains unclear whether these characteristics predated surgery or were exacerbated postoperatively. Longitudinal studies comparing pre- and postoperative gait within the same subjects are warranted.

In the arthrodesis group, no strong correlations were observed between LL and gait parameters, likely due to the limited sample size. Weak correlations suggested an association between reduced LL and increased foot progression angle. Given the multifactorial determinants of foot progression angle [43, 44], these findings should be interpreted cautiously. Larger studies are needed to confirm whether similar relationships exist in patients after lumbar arthrodesis.

Although the present study was not designed to evaluate therapeutic interventions, the observed gait differences highlight the potential value of objective gait assessment in the functional evaluation of patients following lumbar arthrodesis. Balance training, including virtual reality [49], has been shown to improve postural control and gait performance in patients with chronic low back pain and in those following lumbar fusion surgery [50], supporting its inclusion in rehabilitation programs. Custom-made orthopedic insoles have also been shown to improve low back pain in individuals with pronated feet by correcting pelvic tilt and vertebral rotation during gait [9, 10, 35, 36, 38]. Similarly, rocker-bottom shoes may enhance paravertebral muscle activity, promoting spinal extension and reducing pain during daily activities, although postural changes remain unclear [51–53]. Further research is required to evaluate the efficacy of these interventions in degenerative low back pain.

This study has several limitations. The small sample size may have limited statistical power. High BMI influences low back pain prevalence [54] but may also affect palpation accuracy and marker placement during spinal reconstruction. Whereas in this study well-trained examiners applied the A-palp method, it must be acknowledged that examiner experience is critical: inaccurate marker positioning and spinal palpation may introduce bias and affect results. Although the control group was selected to improve comparability with the arthrodesis group in terms of age, sex, and BMI, residual demographic differences may still have influenced gait and spinal curvature measurements. Previous multivariate analyses have suggested that factors such as sex may influence lumbar lordosis independently of age [55]. Furthermore, postoperative clinical data, including pain intensity, disability scores, medication use, and rehabilitation protocols, were not consistently available. These missing variables may have influenced gait performance and limit the clinical interpretation of the observed differences. In addition, the absence of a non-surgical chronic low back pain comparison group limits the ability to determine whether the observed gait characteristics are specifically associated with lumbar arthrodesis or may also reflect gait alterations related to chronic low back pain more generally.

An additional limitation of this study is its cross-sectional design, which precluded assessment of the temporal evolution of gait following lumbar arthrodesis. Consequently, it was not possible to determine whether the observed gait characteristics reflected pre-existing adaptations related to chronic low back pain, postoperative changes induced by surgery, or the effects of rehabilitation. Recent prospective studies have shown that longitudinal three-dimensional gait analysis can detect functional improvements after lumbar fusion and that these changes correlate with clinical outcome measures such as pain and disability scores [56]. Longitudinal studies integrating repeated gait assessment, spinal alignment measurements, and clinical outcomes are therefore warranted to better characterize postoperative functional recovery.

Given the exploratory nature of the correlation analyses and the number of comparisons performed, the observed associations should be interpreted cautiously. Nevertheless, larger studies remain necessary to confirm the observed associations and improve generalizability.

The present findings further support the growing interest in objective gait assessment as a complementary outcome measure after lumbar arthrodesis. While conventional radiographic parameters remain essential to evaluate spinal alignment, quantitative gait analysis may provide additional information regarding functional recovery. Together with recent prospective evidence demonstrating improvements in gait following lumbar fusion [56], our results suggest that combining radiographic, clinical, and biomechanical assessments may provide a more comprehensive evaluation of postoperative outcomes.

## Conclusion

Lumbar arthrodesis remains a last-resort treatment for disabling chronic low back pain of degenerative origin, yet its functional impact is still poorly understood. Our study identified specific differences in gait parameters among patients who underwent lumbar arthrodesis; mean lumbar lordosis and thoracic kyphosis values were numerically lower in the arthrodesis group, although these differences did not reach statistical significance. However, these findings should be interpreted with caution, as the absence of preoperative data and the small sample size do not allow us to determine whether these differences result from the surgical procedure itself or reflect pre-existing gait patterns commonly observed in individuals with chronic low back pain. Future studies with larger cohorts and longitudinal pre- and postoperative assessments are needed to better characterize gait and sagittal spinal alignment patterns following lumbar arthrodesis and guide clinical management strategies.

## Data Availability

All data generated or analysed during this study are included in this published article.
